# Severe spinal cord hypoplasia due to a novel *ATAD3A* compound heterozygous deletion

**DOI:** 10.1016/j.ymgmr.2022.100912

**Published:** 2022-08-24

**Authors:** Tomohiro Ebihara, Taro Nagatomo, Yohei Sugiyama, Tomoko Tsuruoka, Yoshiteru Osone, Masaru Shimura, Makiko Tajika, Keiko Ichimoto, Yuki Naruke, Nana Akiyama, Sze Chern Lim, Yukiko Yatsuka, Kazuhiro R. Nitta, Yoshihito Kishita, Takuya Fushimi, Atsuko Okazaki, Akira Ohtake, Yasushi Okazaki, Kei Murayama

**Affiliations:** a Center for Medical Genetics, Department of Neonatology, Chiba Children's Hospital, Chiba City, Japan; b Department of Neonatology, Japanese Red Cross Fukuoka Hospital, Fukuoka City, Japan; c Center for Medical Genetics, Department of Metabolism, Chiba Children's Hospital, Chiba City, Japan; d Center for Neonatology, Chiba University Hospital, Chiba City, Japan; e Department of Pathology, Chiba Children's Hospital, Chiba City, Japan; f Diagnostics and Therapeutics of Intractable Diseases, Intractable Disease Research Center, Juntendo University, Graduate School of Medicine, Tokyo, Japan; g Department of Life Science, Faculty of Science and Engineering, Kindai University, Osaka, Japan; h Department of Pediatrics and Clinical Genomics, Saitama Medical University, Moroyama, Saitama, Japan; i Center for Intractable Diseases, Saitama Medical University Hospital, Moroyama, Saitama, Japan; j Laboratory for Comprehensive Genomic Analysis, RIKEN Center for Integrative Medical Sciences, Kanagawa, Japan

**Keywords:** ATAD3A, ATPase family AAA-domain containing protein 3A, *ATAD3B*, ATPase family AAA-domain containing protein 3B, *ATAD3C*, ATPase family AAA-domain containing protein 3C, PCH, pontocerebellar hypoplasia, bp, base pairs, mtDNA, mitochondrial DNA, Apgar, an appearance, score, grimace, activity and respiration, SNVs, single nucleotide variants, IUGR, intrauterine growth restriction, PCR, polymerase chain reaction, *RARS2*, arginyl-tRNA synthetase 2, mitochondrial, *SLC25A46*, solute carrier family 25 member 46, MRI, magnetic resonance imaging, Spinal cord hypoplasia, *ATAD3*, Biallelic deletion, Neonate

## Abstract

Biallelic deletions extending into the ATPase family AAA-domain containing protein 3A (*ATAD3A*) gene lead to infantile lethality with severe pontocerebellar hypoplasia (PCH). However, only 12 such cases have been reported worldwide to date, and the genotype–phenotype correlations are not well understood. We describe cases associated with the same novel biallelic deletions of the *ATAD3A* and *ATAD3B/3A* regions in Japanese siblings with severe spinal cord hypoplasia and multiple malformations, including PCH, leading to neonatal death. The ATAD3A protein is essential for normal interaction between mitochondria and endoplasmic reticulum and is important for mitochondrial biosynthesis. The cases were evaluated using whole-genome sequencing for genetic diagnosis of mitochondrial disease. Spinal cord lesions associated with biallelic compound heterozygous deletion extending into the *ATAD3A* gene have not been reported. In addition, the *ATAD3A* deletion was 19 base pairs long, which is short compared with those reported previously. This deletion introduced a frameshift, resulting in a premature termination codon, and was expected to be a null allele. The pathological findings of the atrophic spinal cord showed gliosis and tissue destruction of the gray and white matter. We describe spinal cord lesions as a new central nervous system phenotype associated with a biallelic compound heterozygous deletion extending into the *ATAD3A* gene. Biallelic *ATAD3A* deletions should be considered in cases of mitochondrial disease with spinal cord hypoplasia and PCH.

## 1 Introduction

Disruption of *ATAD3* (ATPase family AAA-domain containing protein 3) cluster, specifically *ATAD3A*, was recently shown to be an important cause of various neurological syndromes. The *ATAD3* cluster, located on chromosome 1p36.33, is composed of three paralogs (*ATAD3A* [MIM:612316], *ATAD3B* [MIM:612317], and *ATAD3C* [MIM:617227]) formed through two tandem segmental duplications [1,2]. The *ATAD3A* gene encodes a mitochondrial transmembrane ATPase that is involved in organizing and replicating mitochondrial DNA (mtDNA) [[3], [4], [5]]. *ATAD3* dysfunction and deficiency cause disturbed mitochondrial morphology and dynamics [6], loss of cristae [7], perturbed mtDNA and cholesterol metabolism [5], impaired mitochondrial steroidogenesis [8], and decreased amounts of mitochondrial oxidative phosphorylation supercomplexes [7,9]. Variants at this locus are associated with a wide phenotypic spectrum, including neonatal death, seizures, contractures, hypotonia, peripheral neuropathy, hereditary spastic paraplegia, optic atrophy, corneal clouding, congenital cataract, and hypertrophic cardiomyopathy, as well as neuroradiology findings such as simplified/delayed sulcal and gyral pattern and pontocerebellar hypoplasia (PCH) [5,6,[9], [10], [11], [12], [13]]. Various genetic factors have been reported at this locus, including de novo or inherited dominant single nucleotide variants (SNVs) [6,10], inherited recessive SNVs [[10], [11], [12]], inherited recessive biallelic deletions [5,10,11,13], and de novo dominant duplication causing cardiac failure just before and after birth [9,14].

The *ATAD3* locus is one of the most common causes of nuclear-encoded pediatric mitochondrial disease; however, the repetitive nature of the locus means *ATAD3* diagnoses may be frequently missed [9]. To date, only 12 cases of biallelic deletions extending into the *ATAD3A* gene have been reported globally(Table 1), and the genotype-phenotype correlations are not well-understood. We describe new cases of novel biallelic deletions of the *ATAD3A* and *ATAD3B/ATAD3A* regions in three Japanese patients associated with severe spinal cord hypoplasia and multiple malformations, which led to their neonatal death. We describe spinal cord lesions with PCH as a new central nervous system phenotype associated with biallelic compound heterozygous deletions extending into the *ATAD3A* gene.

**Table 1:** Clinical findings in cases with biallelic compound heterozygous deletion extending into the *ATAD3A* gene.

	This study			Harel et al. (2016)	Desai et al. (2017)					Peeters-Scholte et al. (2017)				Zheng Yie Yap et al. (2021)	
	II-1	II-2	II-3	F7, II-1	S1a	S1b	S2	S3	S4	S1a	S1b	S3	S4	Family1	Family2
***ATAD3* deletion**	unknown	3B/3A	3B/3A	3C/3B/3A	3B/3A	3B/3A	3B/3A	3B/3A	3B/3A	3B/3A	3B/3A	3B/3A	3B/3A	3C/3B/3A	3B/3A
		38 kbp	38 kbp	68 kb	38 kbp	38 kbp	38 kbp	38 kbp	38 kbp					67 kbp	38 kbp
	3A	3A	3A	3B/3A	3B/3A	3B/3A	3B/3A	3B/3A	3B/3A	3B/3A	3B/3A	3B/3A	3B/3A	3B/3A	3B/3A
	19 bp	19 bp	19 bp	38 kbp	38 kbp	38 kbp	38 kbp	38 kbp	38 kbp					38 kbp	38 kbp
**Age at death**	1 d	20 d	35 d	13 d	5 d	1 d#	5 d	2 d	7 m	1 d	1 d	7 d	5 d	13 d	30 h
**Fetal presentation**															
Polyhydramnios					+		+	+						NR	NR
Early delivery	+	+	+	+	+	+	+	+			+			NR	NR
**Neuroradiology and laboratory findings**															
Cerebellar hypoplasia or atrophy	+	+	ND	+	+		+	+	+	+	+	+	+	+	+
Spinal hypoplasia	+	+	ND												
Elevated plasma or cerebrospinal fluid lactate	+	+	+		+		+	+	+			+	+		+
**Symptoms**															
Respiratory insufficiency	+	+	+	+	+		+	+	+	+	+			+	+
Hypertrophic cardiomyopathy														+	+
Seizures	+	+	+	+				+				+	+	+	+
Contractures	+	+		+	+		+	+	+	+		+		+	–
Congenital cataract/corneal clouding	+	+		+	+					+		+	+	+	+

# Died intrapartum during a lengthy labor, ND = no data, NR = not reported.

## 2 Patient reports

**Fig. 1:**
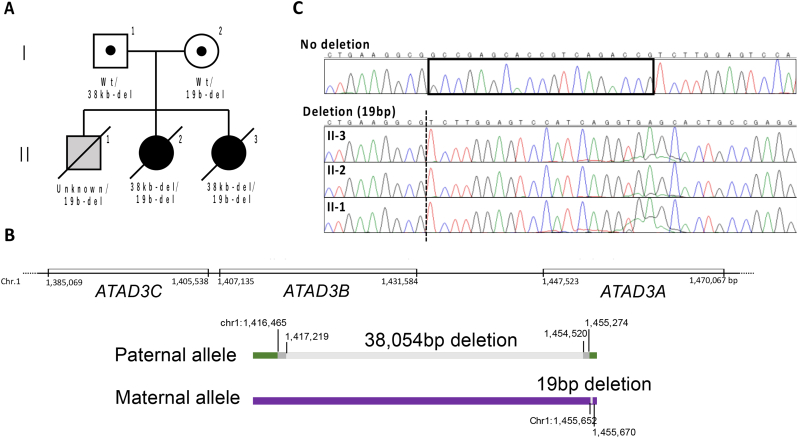
**Pedigree and *ATAD3* genotypes.** (A) Pedigree and ATPase family AAA-domain containing protein 3 (*ATAD3*) genotypes of the family. The paternal deletion in patient II-1 was undetermined because of the low DNA quality (formalin-fixed paraffin-embedded section and dried umbilical cord). del = *ATAD3* deletion; WT = wild-type. (B) Details of *ATAD3* cluster deletion in the paternal allele and *ATAD3A* deletion in the maternal allele. Genomic DNA sequencing of the breakpoint-spanning polymerase chain reaction products revealed the paternal-derived *ATAD3B/ATAD3A* deletion boundaries in each subject, with chromosome 1 coordinates indicated (hg19). Ambiguous regions flanking the deletion boundaries with identical sequences in *ATAD3B* and *ATAD3A* are identified by dark gray boxes. (C) Sanger sequencing of the maternally derived *ATAD3A* 19-bp deletion in the family members II-1, II-2, II-3. The dotted line indicates the location of the deletion, and the square indicates the extent of the 19-bp deletion.

We describe three affected siblings born to healthy Japanese parents who are unrelated by blood. The pedigrees and *ATAD3* genotypes of the family are shown in Fig. 1A. Their clinical features are summarized in Table 1.

Patient II-1 was born in 2005 by emergency cesarean section because of a non-reassuring fetal status at 36 weeks and 4 days of gestation, weighed 2234 g, and had an Apgar score of 2/2. Intrauterine growth restriction (IUGR) was detected at approximately 28 weeks of pregnancy. After birth, resuscitative endotracheal intubation was required for severe neonatal asphyxia. Corneal clouding, low-set ears, joint contractures, seizures, hyperlactacidemia, and metabolic acidosis were observed during intensive care. He died at 15 h after birth from respiratory and circulatory insufficiency. An autopsy was performed. The chromosome (G-banding) was a normal male type, and no further genetic analysis was conducted.

Patient II-2 was born in 2007 by repeat cesarean section at 38 weeks and 1 day of gestation, weighed 2390 g, and had an Apgar score of 2/2. IUGR was detected at 36 weeks of pregnancy. Similar to in Patient II-1, endotracheal intubation was required just after birth; the patient died on day 20 from respiratory and circulatory insufficiency. Corneal clouding, joint contractures, seizures, hyperlactacidemia, metabolic acidosis, and hyperalanemia were observed. A head CT was performed immediately after birth, and postmortem magnetic resonance imaging (MRI) of the head and spinal cord was performed. An autopsy was not approved, but skin and liver biopsies were performed. Mitochondrial respiratory chain enzyme activity in the liver was within normal range. The chromosome (G-banding) was a normal female type. The comparative genomic hybridization array test showed no obvious abnormal findings, and no further genetic analysis was performed.

Patient II-3 was born in 2011 by repeat cesarean section at 38 weeks and 1 day of gestation, weighed 2294 g, and had an Apgar score of 3/2. IUGR was detected during pregnancy. Similar to that in patients II-1 and II-2, endotracheal intubation just after birth was required; the patient died on day 35 from renal and circulatory insufficiency. Seizures, hyperlactacidemia, and metabolic acidosis were observed. An autopsy was not performed, but samples of the liver and myocardium were collected by needle biopsy, and enzyme activity was measured. Only myocardium samples showed decreased activity, leading to the diagnosis of complex I deficiency (% citrate synthase ratio = 4.3%).

### 2.1 Whole-genome sequencing and sanger sequencing analysis

As shown in Fig. 1A, patients II-2 and II-3 had compound heterozygosity with a paternally derived 38 kbp *ATAD3B*/*3A* deletion and maternally derived 19 bp deletion in *ATAD3A* exon 6. The paternal-derived deletion was an *ATAD3B/3A* (NC_000001.10) deletion (g.(1416465_1417219)_(1454520_1455274)del), and that from the mother was an *ATAD3A* (NM_001170535) deletion (c.646_664del:p.Ala216Serfs*235) (Fig. 1B, C). There are no reports of a 19 bp *ATAD3A* deletion in large human genome datasets (gnomAD, GenomeAsia 100 K, jMorp 14KJPN [Japanese multiomics reference panel], Biobank Japan) [[15], [16], [17], [18]]. No other genes with homozygous or compound hetelozygous with potentially pathogenic variants were found in the WGS of patient II-3. In patient II-1, the only specimens available were formalin-fixed paraffin-embedded sections and the dried umbilical cord. DNA extracted from the fixed section was not amplified by polymerase chain reaction (PCR). DNA extracted from the umbilical cord showed no PCR amplification of 5 kbp and no verification of the 38 kbp-del of paternal origin, but a short sequence of 163 bp was amplified by PCR amplification, revealing a deletion of maternal origin. No amplification was detected even in short PCRs of approximately 400 and 600 bp, unrelated to *ATAD3*, which was attributed to the low DNA quality.

### 2.2 Head and spinal imaging findings

**Fig. 2:**
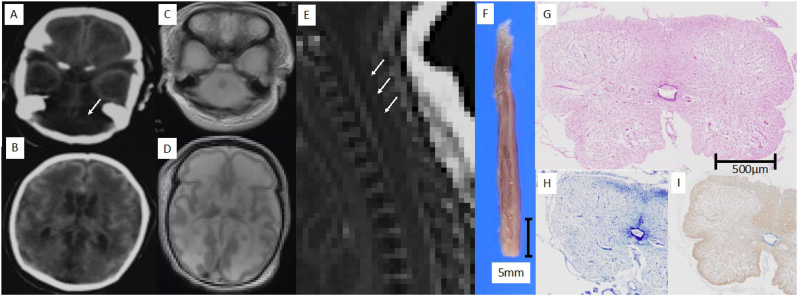
**Imaging and autopsy findings.** (A, B) Head computed tomography (CT) of patient II-2 was performed immediately after birth. (A) Cerebellar and brainstem hypoplasia (long arrow). (B) Diffused hypoabsorption areas in the bilateral cerebral white matter. (C–E) Head and spinal magnetic resonance imaging (MRI) of patient II-2 was performed postmortem. (C, D) Axial T2-weighted image shows strong signals in the cerebellum, brainstem, and most of the cortex above the bilateral tents, suggesting that the patient had undergone encephalomalacia. (E) Sagittal T1-weighted image shows prominent thinning of the spinal cord (long arrow). (F) Appearance of thin spine in patient II-1 (scale bar 500 mm). (G-I) Microscopic findings of spine of patient II-1. (G) The spinal cord is thin, and the white and gray matter is obscure (hematoxylin–eosin staining, scale bar 500 μm). (H) Myelin sheaths are indistinct (Kluver–Barrera stain). (I) Extensive gliosis is demonstrated (immunohistochemistry for glial fibrillary acidic protein).

Only patient II-2 underwent head imaging. Head computed tomography (immediately after birth) showed cerebellar and brainstem hypoplasia and diffuse hypoabsorption areas in the bilateral cerebral white matter. Head MRI (postmortem) revealed a high signal on T2-weighted images, suggesting a history of encephalomalacia in the cerebellum, brainstem, and most of the cerebral cortex. MRI of the spinal cord showed prominent thinning of the spinal cord (Fig. 2A–E).

### 2.3 Autopsy findings

An autopsy was performed on patient II-1. Macroscopically, the spinal cord was thin and < 2 mm in diameter, indicating hypoplasia. In addition, myelin sheaths were obscured, and extensive gliosis due to destruction of the gray and white matter was observed (Fig. 2F–I).

## 3 Discussion

We identified novel compound heterozygous deletions in the *ATAD3A* and *ATAD3B/3A* regions in siblings from a healthy non-consanguineous family associated with severe spinal cord hypoplasia, cerebellar hypoplasia, seizures, contractures, and corneal clouding. Twelve cases of biallelic deletions extending into the *ATAD3A* gene have been reported (Table 1). Two of these cases had a 38-kb *ATAD3B/3A* deletion along with a larger 68-kb *ATAD3C/3A* deletion [10,13]. The other ten had biallelic *ATAD3B/3A* deletions, including five cases with homozygous or compound heterozygous 38-kb deletions [5,11]. These clinical manifestations are quite similar, indicating that biallelic *ATAD3B/3A* deletion is an important cause of severe PCH leading to early death. In our cases, the deletion of *ATAD3A* was for 19 bp, which is short compared with the lengths of deletions reported previously for this locus [5,10,11,13]; however, the short deletion was expected to be a null allele because of introduction of a premature termination codon through a frameshift. In addition to the biallelic large deletions in *ATAD3B/3A*, severe PCH and early postnatal death have been reported in cases of homozygous nonsense and missense mutations of *ATAD3A* [11,12]. Based on previously reported cases, genetic conditions that highly reduce *ATAD3A* expression rather than *ATAD3B* expression are mainly associated with neonatal lethality. Although ATAD3A and 3B proteins were not measured in these cases, deletion of *ATAD3B* was found only in one allele, emphasizing the effect of *ATAD3A* loss. Furthermore, imaging findings of the central nervous system revealed PCH, which is associated with biallelic *ATAD3A* deletions, and spinal cord hypoplasia, which has not been fully described. Spinal cord hypoplasia involves tissue destruction and gliosis of the gray and white matter, with few neurons remaining. The lesion may have developed during a chronic course that began in utero. Neurons depend primarily on mitochondrial oxidative phosphorylation for their energy and have a limited ability to upregulate glycolysis compared to astrocytes and oligodendrocytes [19]. Therefore, neurons are more susceptible to mitochondrial dysfunction, and gliosis likely forms after neuronal destruction.

Spinal cord involvement in patients with mitochondrial disorders is not uncommon and.

has been reported in patients with PCH [20]. PCH is currently classified into 11 types, although *ATAD3A* deletion is excluded [21]. Among these classifications, PCH type 1E [solute carrier family 25 member 46 (*SLC25A46*)] and PCH type 6 [arginyl-tRNA synthetase 2, mitochondrial (*RARS2*)] are derived from the etiologic genes of mitochondrial diseases, and PCH type 1E is characterized by respiratory failure requiring tracheal intubation immediately after birth, congenital contractures, and spinal cord involvement, which is consistent with the clinical findings of *ATAD3A* deletion. However, spinal cord lesions in PCH1 are limited to degeneration and reduction of the anterior horn cells, which differs from the present case, in which tissue destruction of the entire spinal cord was observed, albeit with anterior horn predominance. When PCH is associated with spinal cord hypoplasia, biallelic *ATAD3A* deletions should be considered as differential.

This study had some limitations. *ATAD3A* deletion could only be demonstrated in one allele in the case in which pathological autopsy was performed. Of the two remaining cases in which *ATAD3A* deletion was found in both alleles, spinal cord hypoplasia was confirmed in only one. Therefore, additional cases should be evaluated to clarify the relationship between biallelic *ATAD3A* deletion and spinal cord lesions.

## 4 Conclusions

We reported cases of siblings with severe spinal cord hypoplasia as a new central nervous system phenotype accompanied by neonatal death associated with compound heterozygous deletions in the *ATAD3A* and *ATAD3B*/*ATAD3A* regions. In cases of fetal cerebellar hypoplasia and spinal cord hypoplasia, compound heterozygous deletions in the *ATAD3A* gene must be identified to differentiate the disease and are important for predicting prenatal prognosis.

## Ethics approval

This is an observational retrospective patient report that did not involve any research-based patient intervention. All interventions were intended to diagnose and treat the patient. No aspect of this report contradicts the Helsinki Declaration of 1975, as revised in 2000.

## Funding

This work was supported by the 10.13039/100009619Practical Research Project for Rare/Intractable Diseases from the Japan Agency for Medical Research and Development, AMED [grant numbers JP22ek0109468, 21kk0305015, 21ek0109485, 19ek0109273].

## Patient consent

Written informed consent for the publication of these data was obtained from the patient's mother.

## Author contributions

TE, TT, and KM conceptualized this report, were involved in clinical practice, and drafted the initial manuscript; TN, YS, YO, MS, MT, KI, YN, NA, LS, YY, KN, YK, TF, AO, AO, and YO coordinated and supervised data collection and critically reviewed the manuscript for important intellectual content. All authors approved the final manuscript as submitted and agree to be accountable for all aspects of the work.

## Declaration of Competing Interest

Authors declare no conflict of interest.

## Data Availability

Data will be made available on request.

## Appendix A Supplementary data

Supplementary material: Details of genetic testing methods and results Image 1
