# Supplementary material for: Severe spinal cord hypoplasia due to a novel ATAD3A compound heterozygous deletion
Source: Mol Genet Metab Rep. 2022 Aug 24;33:100912. doi: 10.1016/j.ymgmr.2022.100912 (PMC9428837; doi:10.1016/j.ymgmr.2022.100912)
Supplement: Supplementary file 1 — Supplementary material: Details of genetic testing methods and results [file mmc1.docx]

**Supplementary Material**

**1. Whole-genome sequencing and Sanger sequencing**

Genomic DNA was extracted from the fibroblasts of patient II-3 and blood of the parents. Indexed genomic DNA libraries were prepared from the genomic DNA of patient II-3 and parents using MGIEasy PCR-Free DNA Library Prep Set V1.1 (BGI, Shenzhen, China) according to the manufacturers’ protocols, followed by sequencing on MGISEQ-2000 using MGISEQ-2000RS High-throughput Sequencing Set PE100 V3.0 (BGI). A bioinformatics pipeline was run as previously described [1]. Whole-genome sequencing revealed two types of deletions in the *ATAD3B/ATAD3A* region, which were investigated in all family members using long-range PCR amplification with primers as described previously [2]. PCR products were directly sequenced using BigDye v3.1 Terminators and ABI 3130XL (Applied Biosystems, Foster City, CA, USA). The insert was sequenced following cloning of the PCR product into a plasmid (pCR-Blunt II-TOPO) to confirm the 19-bp deletion derived from the mother. This step was performed because discrimination between *ATAD3A* and *ATAD3B* by the primers was incomplete.

**2. Sequences of primers for the *ATAD3* region on chromosome 1.**

|  | Primer Name | Sequence | Orientation | Region (hg19) | Target | Reference | Product (bp) | FWD+REV |
| --- | --- | --- | --- | --- | --- | --- | --- | --- |
| 1 | OT570-F | 5' - TGTGCCTGCCTTGTTTCATA - 3' | FWD | chr1:1452082-1452101 | 19 bp-del (mo) | [2] | 4,087 | 1+3 |
| 2 | OT572-F | 5' - GAGGGGGTCTTCTTCACAT - 3' | FWD | chr1:1413007-1413025 | 38 kbp-del (fa) | [2] | 43,162 (5,107 if del) | 2+3 |
| 3 | OT473-R | 5' - AGCTTGAGAAGGGAGGAGAAGC - 3' | REV | chr1:1456147-1456168 | 38 kbp-del (fa) 19 bp-del (mo) | [2] |  |  |
| 4 | 19del-F1 | 5' - GAATGAGATGCTGCGAGTGGAGG - 3' | FWD | chr1:1455555-1455577 | 19 bp-del (mo) |  | 163 | 4+5 |
| 5 | 19del-R1 | 5' - TGTGGCCGGCCCGGGCCTC - 3' | REV | chr1:1455699-1455717 | 19 bp-del (mo) |  |  |  |
| 6 | PCR-400b-F | 5’ – TTCCTGCACCATAACCCTCA – 3’ | FWD |  |  |  | 400 | 6+7 |
| 7 | PCR-400b-R | 5’ – GGTTTTGGATCGGTTCATCC – 3’ | REV |  |  |  |  |  |
| 8 | PCR-600b-F | 5’ – AATCATACAAAGCCCCCGCA – 3’ | FWD |  |  |  | 598 | 8+9 |
| 9 | PCR-600b-R | 5’ – GAGATGTTGGATGGGGTGGG – 3’ | REV |  |  |  |  |  |

**3. Genomic DNA sequencing to map the breakpoint junction of *ATAD3A***

Red band indicates the deletion site in the *ATAD3B/3A* region that is detected by CNVnator [3]. The blue line between the red arrows indicates paired reads showing the deletion.

[1] Y. Kishita, M. Shimura, M. Kohda, et al., Genome sequencing and RNA-seq analyses of mitochondrial complex I deficiency revealed Alu insertion-mediated deletion in *NDUFV2*, Hum Mutat. 42 (2021) 1422–1428. https://doi.org/[10.1002/humu.24274](https://doi.org/10.1002/humu.24274).

[2] R. Desai, A.E. Frazier, R. Durigon, et al. *ATAD3* gene cluster deletions cause cerebellar

dysfunction associated with altered mitochondrial DNA and cholesterol metabolism. Brain. 140 (2017) 1595–1610. <https://doi.org/10.1093/brain/awx094>.

[3] Abyzov A, Urban AE, Snyder M, et al. CNVnator: an approach to discover, genotype, and characterize typical and atypical CNVs from family and population genome sequencing. Genome Res. 21 (2011) 974-84. https://doi.org/10.1101/gr.114876.110.
